# Wilson’s Disease: Diagnosis in Novel Way

**DOI:** 10.7759/cureus.18650

**Published:** 2021-10-11

**Authors:** Pankaj K Kannauje, Vinay R Pandit, Preetam N Wasnik, Pranita Das, Nanditha Venkatesan

**Affiliations:** 1 General Medicine, All India Institute of Medical Sciences, Raipur, IND

**Keywords:** wilson’s disease, copper, chronic liver disease, ceruloplasmin, kayser-fleischer ring

## Abstract

Wilson’s disease first described by Kinnier Wilson in 1912, is a rare autosomal recessive genetic disorder involving a defect in copper metabolism. This disease affects between one in 30,000 to one in 100,000 individuals and has a carrier frequency of one in every 90. It is characterized by hepatic and neurological symptoms. The usual age of presentation is 4 to 40 years but this disorder has been detected in children as young as three years and adults as old as 70 years with males and females being equally affected. Diagnosing Wilson’s disease at the earliest is crucial as it is not only progressive and fatal if untreated, but also responds promptly to medication. Here we are going to present a novel way to diagnose a case of Wilson disease in a resource-limited setting. The diagnosis was possible with detailed present and past history raising strong clinical suspicion of environmental or genetically related disease. The diagnosis was done in a novel way by first diagnosing in daughter thereafter confirming the same diagnosis in patient.

## Introduction

Wilson's disease is a genetic disorder and its symptoms are primarily due to the accumulation of excess copper in the liver caused by reduced copper excretion in bile. this disease can present as both hepatic or neurological manifestation. While the incidence of Wilson’s disease is relatively rare, it shows an increase in areas with consanguineous marriages. Wilson’s disease is rare however being in a rich mineral state, we have seen various presentations of Wilson’s disease but most cases are not reported. Here we describe a novel way to diagnose a patient who was un-cooperative and could not afford the investigation of Wilson’s disease.

## Case presentation

A 50-year-old female patient was brought to the emergency department of our hospital by her two young daughters. Patient was crying furiously, shouting gibberish atop her voice. The daughters revealed that this was a common occurrence and noted that the patient had abnormal posturing, which was corroborated by generalized rigidity as confirmed by examination.

On neuroimaging, head computed tomography revealed multiple infarcts, indicating cerebrovascular attack. On further enquiry, the daughters reported observing a few cases of rigidity in their hometown with similar behaviors.

**Figure 1:**
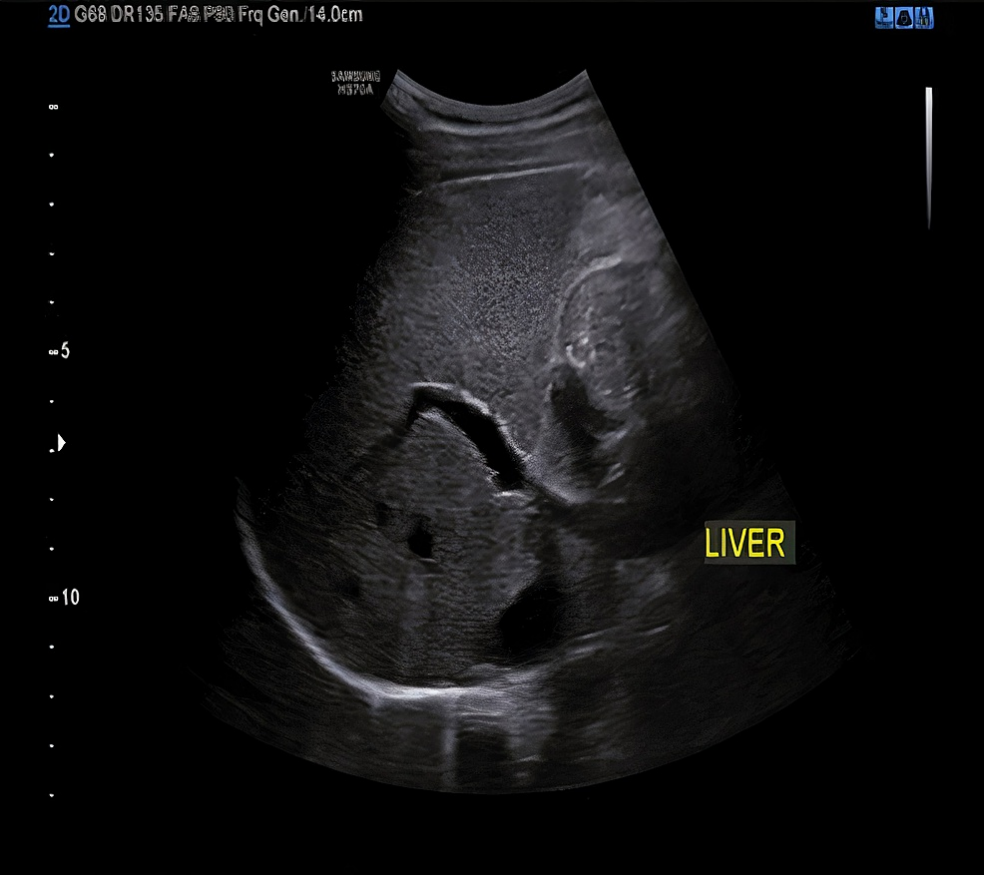
Ultrasound abdomen showing normal liver size and echotexture.

With this vital information, diseases with environmental exposure and geographical location were considered, and Wilson’s disease was considered a differential diagnosis. To rule out Wilson’s disease, we have done routine investigations and recommended serum ceruloplasmin, 24h urinary copper test, and liver biopsy for confirmation. However, owing to financial constraints, the daughters reluctantly refused the aforementioned tests. We decided to look for the Kayser-Fleischer (KF) ring in the patient, but she was uncooperative, making it almost impossible to test her. Her Hemoglobin was 10.8 g/L, platelets 178 x109/L, WBC count of 6.06x109/L. Her liver function test showed normal bilirubin level with serum glutamic-oxaloacetic transaminase (SGOT)-24 IU/L, serum glutamic pyruvic transaminase (SGPT)-32 IU/L, alkaline phosphate- 110 U/L, total protein 6.2 gm/dL, and albumin 4.1 g/dL. Her prothrombin time (PT) was 12.2 seconds, international normalized ratio (INR 1.1), and activated partial thromboplastin time (aPTT) of 29.5 seconds. Her ultrasound abdomen showed normal liver size (10.8 cm) and normal portal vein diameter (8 mm) without any evidence of cirrhosis (Figure 1).

**Figure 2:**
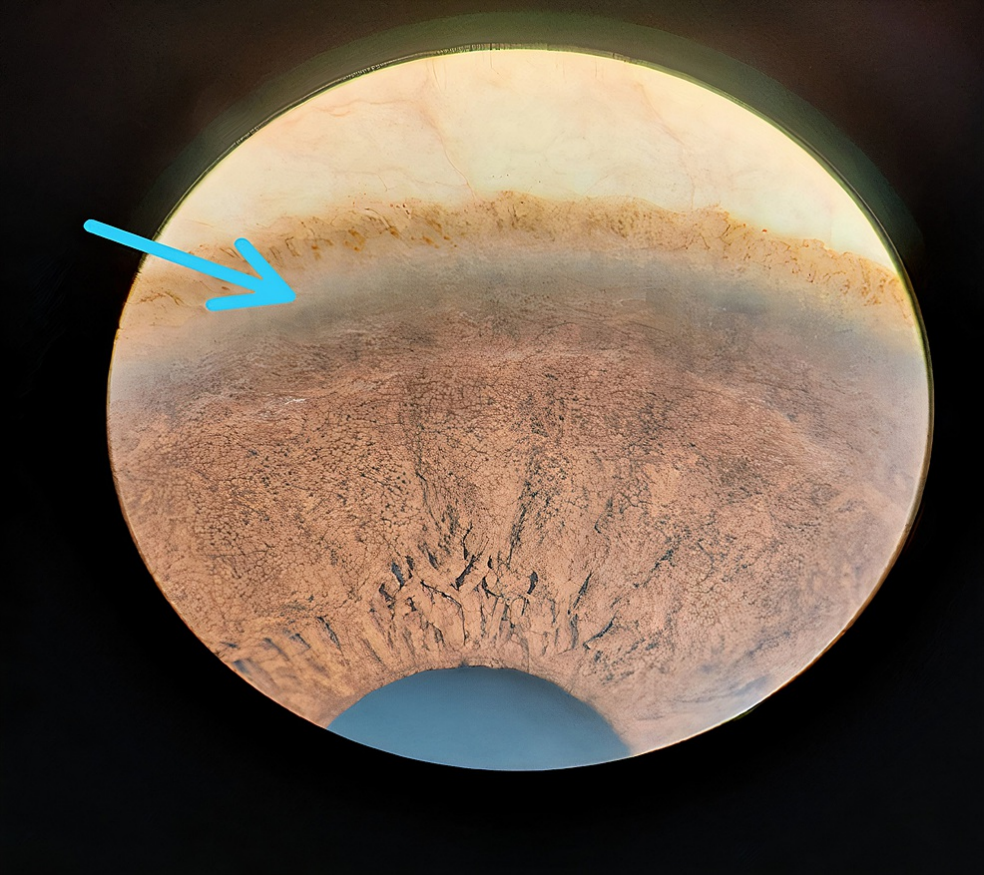
KF ring (in daughter). KF:  Kayser-Fleischer.

Considering environmental exposure and genetic predisposition, we planned to look for KF ring in the daughters. Any form of genetic pigmentation was ruled out, and the elder daughter subsequently tested positive for the KF ring (Figure 2).

Using that as leading evidence, we shared the cost of 24h urinary copper test for the patient and her elder daughter, and the level of copper in both individuals was markedly high, confirming our suspicion. The Leipzig score was calculated for the patient and it was >4 confirming the diagnosis of Wilson. The patient was started on D-penicillamine (500 mg), aspirin (75 mg), and zinc (100 mg). The patient drastically improved, and after staying for three weeks she was discharged. The patient and elder daughter are on regular follow-up and doing well.

## Discussion

Wilson’s disease is caused by mutations in the ATP7B gene, which regulates the protein transporters that excrete excess copper via bile [1,2]. The lack of adequate excretion and accumulation of excess copper in the blood lead to oxidative stress and damages to the cells, leading to clinical manifestations [3].

As the disease affects the liver and basal ganglia, symptoms usually are primarily related to the liver and brain. Liver-related signs and symptoms include Jaundice, itching, abdominal pain, generalized weakness, vomiting, and ascites. Neurological signs and symptoms include tremors, chorea, personality changes, depression, anxiety, headaches, insomnia, seizures, hallucinations, ataxia, and mask-like facies.

On examination, the stigmata of chronic liver disease are often observed, if the disease has progressed. Slit-lamp examination will reveal KF rings on the cornea. Moreover, skeletal involvement, similar to premature osteoarthritis and arthropathy, is common. Hemolytic anemia, Fanconi syndrome-like symptoms and urolithiasis are also observed in some patients.

For clinical suspicion, examining the serum levels of ceruloplasmin is recommended as the first-line diagnostic test. Values of less than 20 mg/dL (normal 20-40 mg/dL) favor Wilson’s disease but can be normal in few cases. However, it is prudent to consider that low ceruloplasmin levels may be observed in nay protein-deficiency order. Urinary copper levels higher than 100 µg/dL indicate excess copper in the circulation. These two diagnostic tests along with KF ring are adequate to diagnose Wilson’s disease. However, the gold standard for diagnosis remains liver biopsy to determine liver copper levels. A positive result is a copper level of greater than 250 µg/g dry liver tissues [4].

Magnetic resonance imaging (MRI) is required for cases with brain involvement. MRI T2-weighted sequence may show hyperintensities in the basal ganglia and demonstrate the characteristic “face of the giant panda” pattern. Screening of first and second-degree relatives is advised.

Differential diagnoses include conditions causing chronic active hepatitis, primary biliary cirrhosis and hemolytic anemias due to various causes. Neuropsychiatric disorders that can be mistaken include various types of parkinsonian syndromes, pantothenate kinase deficiency associated with neurodegeneration (iron accumulation), neuroacanthocytosis syndromes, and Huntington’s disease among others. These conditions may present with personality changes, rigidity, dystonia, and movement disorders and cause confusion during diagnosis.

Due to difficulties in diagnosing Wilson’s disease, the Leipzig (Ferenci) score was devised by a group of experts to help diagnose patients (Table 1).

**Table 1:** Leipzig score: adapted from scoring system developed at the 8th international meeting on Wilson's disease [5].

Typical clinical signs & symptoms		Other tests	
KF ring		Liver copper (in the absence of cholestasis)	
Present	2	>5x ULN (>4µmol/g)	2
Absent	0	0.8-4 µmol/g	1
Neurologic symptoms		Normal <0.8 µmol/g	-1
Severe	2	Rhodanine positive granules	1
Mild	1		
Absent	0		
Serum ceruloplasmin		Urinary copper (in the absence of hepatitis)	
Normal (>0.2g/L)	0	Normal	0
(0.1-0.2 g/L)	1	1-2x ULN	1
(<0.1 g/L)	2	>2x ULN	2
		Normal, but >5x ULN after D- penicillamine	2
Coombs negative Hemolytic Anaemia		Mutation analysis	
Present	1	On both chromosomes detected	4
Absent	0	On 1 chromosomes detected	1
		No mutation detected	0
Total score		Evaluation	
4 or more		Diagnosis established	
3		Diagnosis is possible, more test needed	
2 or less		Diagnosis very unlikely	

It comprises many key investigations for making the diagnosis of Wilson’s disease and applies to the adult population [5]. The Leipzig score was calculated for our patient and it was more than 4 confirming our diagnosis. The mainstay therapy for Wilson’s disease is copper chelation therapy with D-penicillamine and trientine. Trientine is preferred because of fewer side effects. Oral zinc may also be administered as it competes with copper for absorption at metallic ion transporter sites [6,7].

Tetrathiomolybdate (TM) is preferred over zinc in case of neurological involvement but availability and high cost are a challenge in resource-poor settings. It is vital to educate the patient on the adverse effects of chronic chelation therapy, which can make symptoms worse.

For muscle rigidity, spasticity, and parkinsonian features, muscle relaxants, including baclofen, anticholinergic (trihexyphenidyl), gamma-aminobutyric acid antagonists, and levodopa, may be used.

Patient education regarding dietary modifications, such as avoidance of hepatotoxic medications, alcohol, and diet rich in copper including mushrooms, chocolate, nuts, dried fruits, liver, and shellfish, should be emphasized. As copper-chelating treatments take up to six months to start working, physiotherapy and occupational therapy are beneficial in the neurological form of the disease.

Long-term monitoring is essential with measurements of urinary copper, complete blood counts, free copper, and renal and liver function at 4- to 8-week intervals. Most patients need lifelong chelation therapy with follow-up. If untreated, the disease will eventually lead to chronic liver disease and may even progress to hepatocellular carcinoma in some cases. Genetic counseling is necessary to prevent the transmission of the defective gene.

## Conclusions

Wilson’s disease is a Genetic disorder that can have a variety of presentations and can be controlled if detected early. The treatment modality is lifestyle modification, proper follow-up, and physiotherapy. If disease control is not achieved after initial treatment, titrating the dose of chelating agent is indicated to prevent the damage of the internal organs.
